# Brazos Valley Medical Association

**Published:** 1900-12

**Authors:** W. B. Briggs

**Affiliations:** Secretary Brazos Valley Medical Association


### Society Notes.

### Brazos Valley Medical Association.

The Brazos Valley Medical Association of Texas convened in the city of Cameron on the 13th and 14th of November, 1900, and was called to order by the president, Dr. J. P. Oliver, of Caldwell, at the Auditorium at 2 p. m.

[The program as announced in our November number was carried out.—Ed.]

Addresses of welcome were delivered by the mayor, J. M. Ralston, and Dr. M. K. Scott. Dr. J. P. Oliver, the president, responded as follows:

*Ladies and Gentlemen, Fellows of the Brazos Valley Medical Association, Gentlemen of the Committee of Arrangements:*

It affords me a great deal of pleasure to have the happy privilege of responding to the eloquent addresses of welcome which have just been made on behalf of the fellows of the Brazos Valley Medical Association to return our thanks to the distinguished gentlemen who made them, and to those whom they represent, for their warm and cordial words of welcome. We have met in your beautiful and progressive city as students of science, and the representatives of one of the best professions ever known to mortal man. It matters not where the victim falls, whether on the tented fields of carnage, in the hovel of the poor, on the public highways, in the palaces of the opulent or among the pestilential epidemics, there is one soldier who stands as firm as the rocks of Gibraltar at his post of duty, and that soldier is the true physician. He, too, may fall a victim to the same influences which he is combatting, but his sword of duty is never sheathed until God calls him home. The hospitality of your beautiful city is not less proverbial than the beauty and refinement of her ladies. Beautiful city, peerless professional and business men—no wonder her women are pure and fair, and her men brave and chivalrous. Again we return to you our thanks for your warm words of welcome, and promise to honor that welcome.

More interest was manifested at this meeting than at any previous one. The discussions were more thorough, and it was with feelings of regret that we were forced to postpone further deliberations. Several papers remain on hand for the next convention.

Calvert was selected as the next place of meeting—the second Tuesday and Wednesday in May, 1901.

The musical entertainment on the night of the 13th was delightful; the sumptuous banquet on the evening of the 14th, tendered the association by the citizens, was highly enjoyable, and the X ray exhibitions could not be surpassed. In the large hall of the Auditorium there were assembled over two hundred invited guests, and the menu was most excellent and ample. Cameron will not be forgotten.

The following toasts were responded to:

1. "The Brazos Valley Medical Association," Dr. J. P. Oliver.
2. "Ye Handmaids of Medicine," Dr. R. S. Carroll, Calvert.
3. "Ye Highwayman," John Sharp, Cameron.
4. "Our Victims," Prof. J. E. Thompson, Galveston.
5. "The Doctor's Reward," Dr. W. B. Briggs, Easterly.
6. "The Doctor's Wife," Monta J. Moore, Cameron.
7. "Salmagundi," Dr. R. W. Nobles, Temple.
8. "The Doctor X Rayed," Dr. A. C. Scott, Temple.
9. "The Doctor of the Old School," Rev. C. C. Weaver, Cameron.

Dr. A. C. Scott said that he had examined only one doctor through the X ray, and that he found nothing in him. (The examination took place *before* the banquet.)

Judge R. B. Pool, as toastmaster, was a most excellent selection. The responses were highly enjoyed.

The following resolutions were unanimously adopted:

"WHEREAS, It has been our pleasure to hold the tenth semi-annual session of our association in the city of Cameron; and

"WHEREAS, Nothing has been left undone to insure us both profit and pleasure; therefore, be it

"*Resolved,* That we extend our hearty thanks to the physicians and citizens for their cordial entertainment.

"*Be it further resolved,* That we especially wish to express our appreciation to the ladies of Cameron for the excellent musicale, sumptuous benquet and for their presence, all of which added so much to the pleasure of our stay.

"*Be it further resolved,* That we extend our thanks to Drs. Shaw, Greer and Ferguson for their kind invitation to visit and make headquarters at their pleasant private hospital, which is, indeed, a compliment to their enterprise.

"*Be it resolved* by the B. V. M. A., That we extend our sincere thanks to Dr. A. C. Scott, of Temple, for the X ray demonstrations, which was a profitable treat to us, as well as an indication of his interest in the progress and welfare of our association."

At a late hour the happy occasion was brought to a close, the good-byes were spoken and the visiting physicians departed for their homes, having in their hearts a warm place for the good people of Cameron.

W. B. Briggs,
Secretary Brazos Valley Medical Association.

